# A carbon–carbon hybrid – immobilizing carbon nanodots onto carbon nanotubes† ‡
†Electronic supplementary information (ESI) available. See DOI: 10.1039/c5sc02728d

‡This article is dedicated to David Schuster on the occasion of his 80th birthday.


**DOI:** 10.1039/c5sc02728d

**Published:** 2015-08-20

**Authors:** Volker Strauss, Johannes T. Margraf, Timothy Clark, Dirk M. Guldi

**Affiliations:** a Friedrich-Alexander-Universität Erlangen-Nürnberg , Department of Chemistry and Pharmacy & Interdisciplinary Center for Molecular Materials (ICMM) , Egerlandstrasse 3 , 91058 Erlangen , Germany . Email: dirk.guldi@fau.de ; Fax: +49-9131-852-8307; b Computer-Chemie-Centrum & Interdisciplinary Center for Molecular Materials (ICMM) , Friedrich-Alexander-Universität Erlangen-Nürnberg , Nägelsbachstr. 25 , 91058 Erlangen , Germany

## Abstract

The thrust of this work is to integrate small and uniformly sized carbon nanodots (CNDs) with single-walled carbon nanotubes (SWCNT) of different diameters as electron donors and electron acceptors, respectively, and to test their synergetic interactions in terms of optoelectronic devices. CNDs (denoted *p*CNDs, where *p* indicates pressure) were prepared by pressure-controlled microwave decomposition of citric acid and urea. *p*CNDs were immobilized on single-walled carbon nanotubes by wrapping the latter with poly(4-vinylbenzyl trimethylamine) (PVBTA), which features positively charged ammonium groups in the backbone. Negatively charged surface groups on the CNDs lead to attractive electrostatic interactions. Ground state interactions between the CNDs and SWCNTs were confirmed by a full-fledged photophysical investigation based on steady-state and time-resolved techniques. As a complement, charge injection into the SWCNTs upon photoexcitation was investigated by ultra-short time-resolved spectroscopy.

## Abstract 2

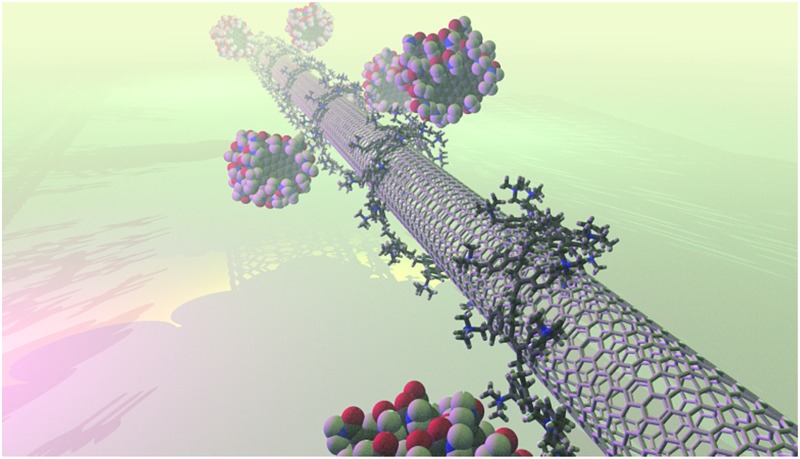
The thrust of this work is to integrate small and uniformly sized carbon nanodots (CNDs) with single-walled carbon nanotubes of different diameters as electron acceptors and electron donors, respectively, and to test their synergetic interactions in terms of optoelectronic devices.

## Introduction

Carbon nanomaterials have emerged as important building blocks for a variety of applications that range from fiber-reinforced polymer composites to nano-electronic devices.^1–5^ This is because they exhibit almost unprecedented potential in terms of structural design and tunable features. Especially in the area of nano-electronics, the opportunities provided by carbon compounds are essentially unlimited.^6–9^ Economically and environmentally, carbon nano-materials offer significant advantages over toxic and rare inorganic materials. Despite the enormous progress made in the area of nano-electronics, there is still a need for further improvements in, for example, device performance.^10^

The rational synthesis of carbon nano-materials suffers from drawbacks in process-upscaling, reproducibility, and homogeneity.^11^ The use of single-walled carbon nanotubes (SWCNTs) in, for example, nano-electronics is hindered by a number of factors that include broad structural distributions and lack of solubility. Their functionalization or hybridization by covalent or non-covalent means is essential for making stable SWCNT dispersions. Major breakthroughs in dispersing SWCNTs have been achieved with the help of non-ionic and ionic surfactants, polymer/oligomer wrapping,^12,13^ and *via* non-covalent functionalization with electroactive molecules.^1,14–18^

As a result, some of the newly prepared SWCNT hybrid materials feature superior light harvesting, charge separation or charge transport properties compared to pristine SWCNTs.^19^ It is especially important that such a non-covalent approach enables SWCNT optoelectronic devices to be made without perturbing the SWCNT's electronic structure.^20–22^ All of these considerations mean that the rational design of hybrid architectures with building blocks designed to perform specific functions is the ultimate goal of many research efforts.^22,23^

In nanoelectronic devices, SWCNT doping by electron donors or acceptors and selective chemical functionalization have proven to be effective ways to enhance specific functions of SWCNTs.^14,15,24^ For example, photosensitization with panchromatic light-harvesters has been shown to be a promising strategy for efficient solar energy conversion.^23,25–27^ Carbon nanodots (CNDs) represent an interesting type of sensitizer in this context. They consist of stacked sp^2^-hybridized carbon layers with functional groups such as carboxy-, hydoxy-, or amide at the periphery.^7,28^ These nanostructures are known for their tunable light absorption and strong, excitation-dependent photoluminescence. Recently, we demonstrated that CNDs can act as either electron donors or acceptors, depending on their counterpart.^29^ All these properties render CNDs ideal candidates for the design of electro- and photoactive hybrids for use in optoelectronic devices.

We now describe the design, synthesis, and characterization of SWCNT/*p*CND hybrid materials, in which SWCNTs function as charge transporters, and *p*CNDs (CNDs prepared by pressure-controlled microwave decomposition of citric acid and urea) as charge dopants. The former are known to exhibit extremely high charge carrier mobilities,^30^ while the latter have broad absorption cross sections combined with strong fluorescence and high photostability.^29^ A simple and straightforward approach to SWCNT/*p*CND hybrid materials based on non-covalent hydrophobic and electrostatic forces was selected. First, hydrophobic forces are used to wrap SWCNTs with positively charged poly(4-vinylbenzyl trimethylamine) (PVBTA), as shown in Fig. S1.† This creates positively charged binding sites on the surfaces of the SWCNTs. Electrostatic forces are then used to immobilize negatively charged *p*CNDs on the SWCNTs. The resulting SWCNT/PVBTA and SWCNT/PVBTA/*p*CND hybrid materials have been fully characterized with particular emphasis on ground- and excited-state interactions between the SWCNTs and *p*CNDs.

## Results and discussion

### Characterization of SWCNT/PVBTA hybrids

**Fig. 1:**
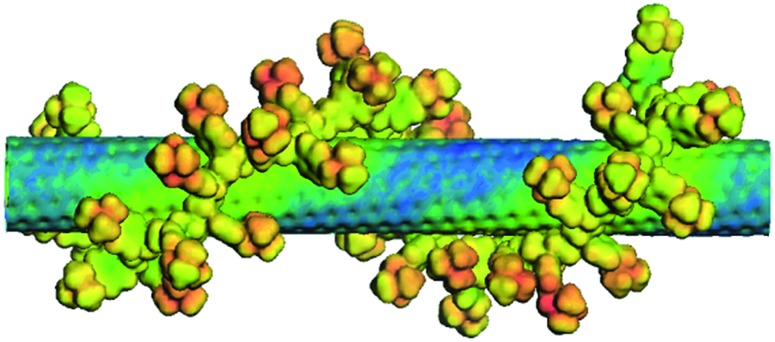
Plot of the molecular electrostatic potential on a 0.03 e^–^ Å^–3^ electron density isosurface of the SWCNT/PVBTA system with Cl^–^ counterions. Color scale is from 1830 (blue) to 1950 kcal mol^–1^ (red).

PVBTA-wrapped SWCNTs have proven to be an ideal platform for immobilizing a variety of nanoparticles.^31,32^ Here, the polymer shell functions as a docking site for negatively charged groups of the nanoparticles. In Fig. 1, the molecular electrostatic potential (MEP) of an SWCNT/PVBTA model system is shown (see computational details). When in contact, the cationic polymer induces a more positive MEP on the SWCNT.

We have used two different types of commercially available SWCNTs, namely CoMoCAT^33^ and HiPCO^34^ SWCNTs, to probe the influence of SWCNT diameter. Initially, the SWCNT/PVBTA hybrid materials were characterized and compared to sodium dodecylbenzenesulfonate (SDBS) suspended SWCNTs as a SWCNT reference. Here, spectroscopic characterizations by both steady-state and time-resolved absorption and emission spectroscopy together with Raman spectroscopy were complemented by microscopic characterization with transmission electron microscopy (TEM).

In Fig. S2,† the absorption spectra of HiPCO SWCNT dispersed with the aid of SDBS and PVBTA in D_2_O are depicted in black and red, respectively. The red-shifts of the S_11_ transitions in the range from 1050 to 1400 nm are most striking in the PVBTA compared to the SDBS sample. Maxima at 1122, 1176, 1202, 1250, and 1267 nm, which are assigned to (8,4), (12,1), (11,3), (9,5), and (8,7) SWCNTs, respectively, appear red-shifted by 20–30 nm relative to their original positions. An appreciable broadening in the absorption spectra of the SWCNT/PVBTA hybrids is evident. Also, the S_22_ transitions in the range between 500 and 800 nm are shifted from, for example, 652 and 735 nm to 658 and 740 nm, respectively. Similar trends in terms of shifting and broadening were published in previous studies.^31,32^ Shifts due to differences in background, light scattering, *etc.* are, however, ruled out.

**Fig. 2:**
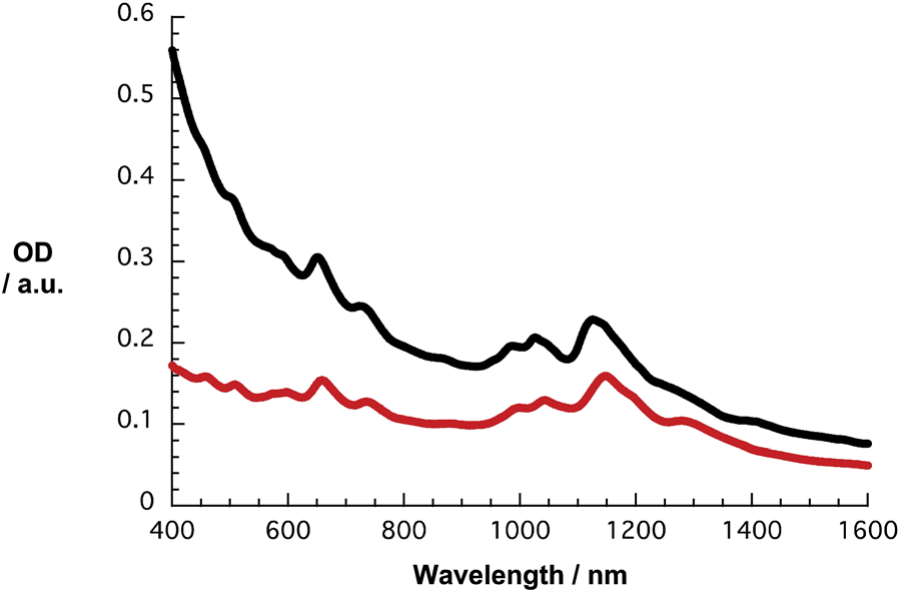
Absorption spectra of CoMoCAT SWCNT dispersed with SDBS (2 wt%, black) and PVBTA (2 wt%, red) in D_2_O.

Likewise, the S_11_ and S_22_ transitions in the CoMoCAT SWCNTs shift to longer wavelength (Fig. 2) for the PVBTA-stabilized samples. However, the S_11_ transitions are shifted less than for HiPCO SWCNTs. For example, the (7,5)-related S_11_ transition at 1125 nm is shifted by 23 nm to 1148 nm and appears more distinctly pronounced in CoMoCAT SWCNT/PVBTA when compared to the SWCNT/SDBS reference. We conclude that this indicates a high degree of debundling of the SWCNTs.^35^

**Fig. 3:**
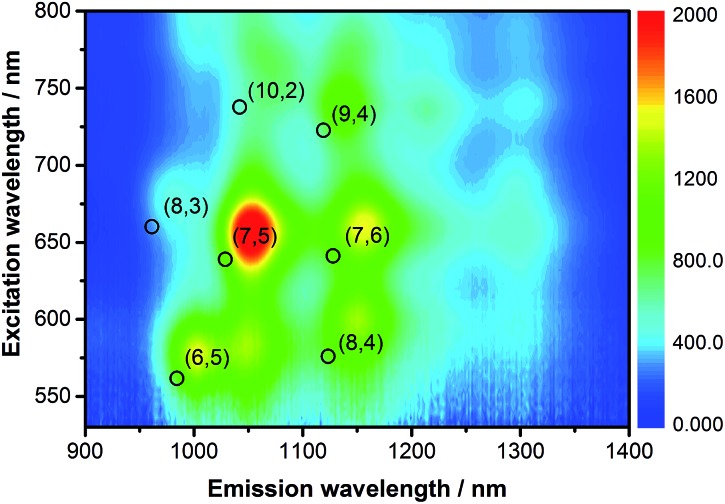
3D-photoluminescence plot of CoMoCAT SWCNT/PVBTA in D_2_O at room temperature. The black circles indicate the positions of the respective features in SWCNT/SDBS in D_2_O.

The emission features of SWCNT/PVTBA were also compared with those of SWCNT/SDBS. As a representative example, the 3D photoluminescence of CoMoCAT SWCNTs dispersed with PVBTA is shown in Fig. 3. The corresponding 3D photoluminescence plots of HiPCO SWCNT in SDBS or PVBTA are shown in Fig. S5 and S6.† At first glance, a striking intensity variation is discernable. Fig. 3 and S4–S6† indicate a preferential dispersion of larger diameter SWCNTs within the samples. For CoMoCAT SWCNT/PVBTA, the most dominant emission corresponds to that stemming from (7,5) and (7,6) SWCNTs, while for HiPCO SWCNT/PVBTA, emission from (7,6), (8,6), and (8,7) prevails. Upon a closer look, red-shifts of the peak positions of at least 25 nm are noticeable – the black circles in the 3D-PL plot indicate the peak positions of the reference dispersion in SDBS – see also Fig. S4–S8.† In particular, the (7,5) and (7,6) peaks in the CoMoCAT species are shifted from 1036 and 1131 nm to 1059 and 1158 nm, respectively. Likewise, the (9,4), (8,6), and (8,7) peaks in the HiPCO samples are shifted from 1109, 1176, and 1265 nm to 1135, 1209, and 1300 nm, respectively. Additional information regarding the quenching of specific SWCNTs present in the samples is given in a statistical analysis in Fig. S9.† To this end, the strongest quenching was observed for (8,4)-SWCNTs.

**Fig. 4:**
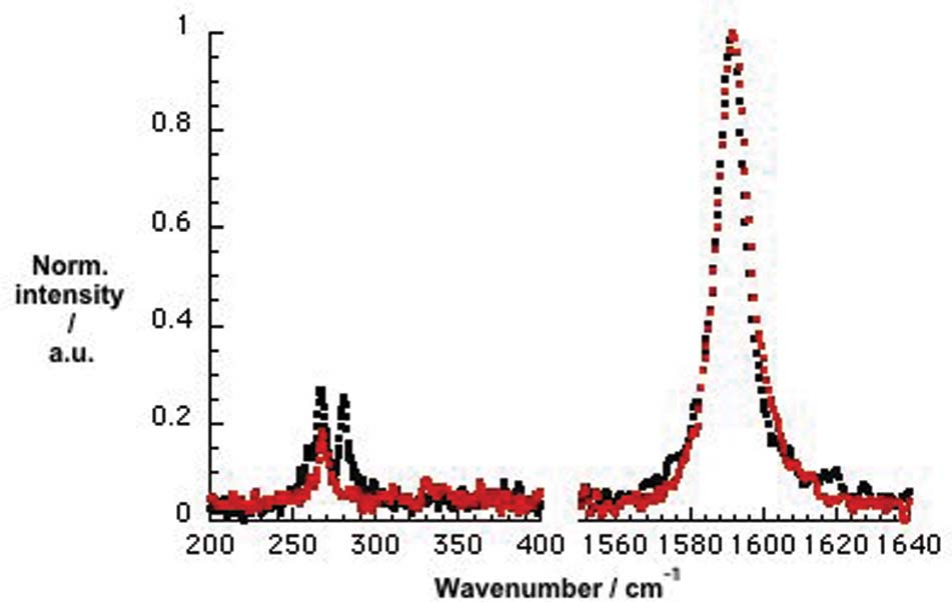
Normalized solid state Raman spectra (*λ*_ex_ = 1064 nm) of CoMoCAT SWCNT/SDBS (black) and CoMoCAT SWCNT/PVBTA (red) with particular emphasis on the RBM- (left), D-band (center) and G-band (right) regions.

To address selectivity towards specific SWCNTs we conducted Raman experiments, in which we compared SWCNT/SDBS references with SWCNT/PVBTA hybrids, as shown in Fig. 4. In the CoMoCAT SWCNT/SDBS reference, two radial breathing modes (RBMs) are visible at 266 and 281 cm^–1^. These are assigned to (7,5) and (7,6) SWCNTs. In CoMoCAT SWCNT/PVBTA, only the (7,5)-related RBM is detected at 267 cm^–1^. The lack of (7,6)-related RBM is due to the shifting of the resonance energy. Moreover, the comparison of CoMoCAT SWCNT/SDBS and CoMoCAT SWCNT/PVBTA reveals a slight upshift of the G^+^-band. In general, this is interpreted as charge injection into the valence band of SWCNTs.^36^ Thus, wrapping with PVBTA moves positive charges close to the SWCNT surface. Complementary experiments with HiPCO samples also imply hole injection, as shown in Fig. S10.†

Transmission electron microcopy of CoMoCAT and HiPCO SWCNTs dispersed with PVBTA was used to investigate the quality of the dispersions in terms of homogeneity and individualization. To this end, SWCNT/PVBTA was applied on a Lacey carbon grid and micrographs were taken in different areas of the grid. Representative medium magnification images shown in Fig. S11 of the ESI† reveal well individualized SWCNTs with lengths of a few hundred nanometers. Notably, only a small number of bundles, each containing a few SWCNTs, were found.

### Characterization of SWCNT/PVBTA/*p*CND hybrids

**Fig. 5:**
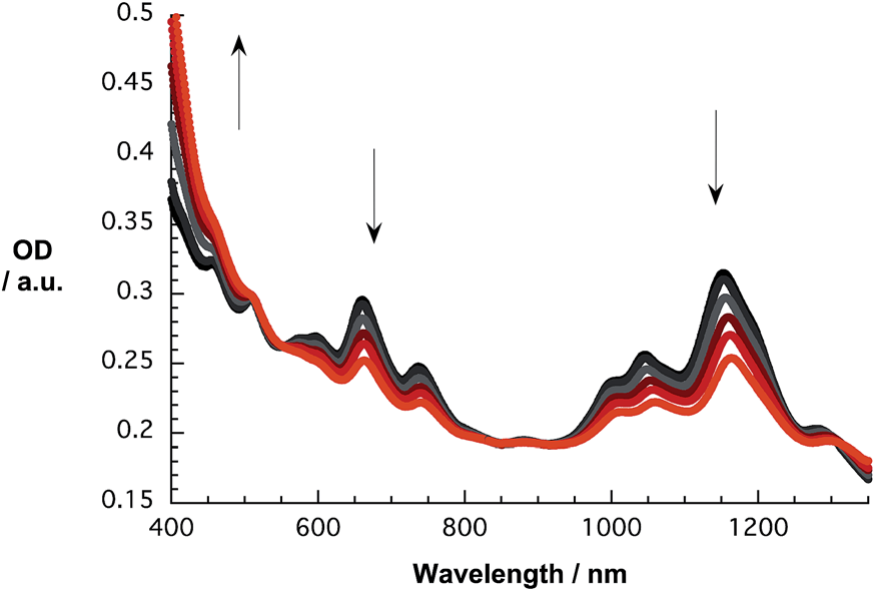
Absorption spectra recorded over the course of sequential addition of *p*CND to CoMoCAT SWCNT/PVBTA in D_2_O at room temperature – arrows refer to the course of addition.

Initial investigations of the interactions between SWCNT/PVBTA and *p*CND were aimed at absorption titration assays, in which the SWCNT/PVBTA concentrations were kept constant and variable concentrations of *p*CND were added stepwise. Here, SWCNT/PVBTA suspensions with optical densities of approximately 0.2 at a given reference point were used – see Experimental section for details. Fig. 5 shows a sequence of absorption spectra of CoMoCAT SWCNT/PVBTA in the presence of increasing *p*CND concentrations – with absorptions in the 300 to 400 nm range – from black to red. The large shifts of the S_11_ transitions in the near infrared are striking. For example, (6,5)-, (7,5)-, and (8,4)-related peaks reveal 12 to 15 nm shifts from 1045, 1152, and 1281 nm to 1058, 1164, and 1296 nm, respectively. Even the S_22_ absorption bands of (7,5)/(7,6) and (8,7) are noticeably shifted from, for example, 660 and 736 nm to 663 and 740 nm, respectively. Considering the aforementioned changes in concert and the fact that SWCNT/PVBTA concentrations were kept constant, we infer a substantial shift of charge density between the SWCNTs and *p*CNDs.

Absorption titrations of HiPCO SWCNT/PVBTA with *p*CNDs reveal qualitatively similar trends. Fig. S12† shows the absorption spectra of HiPCO SWCNT/PVBTA and SWCNT/PVBTA/*p*CND in black and red, respectively. Several maxima in the near-infrared region, for example, at 1149, 1196, 1286 nm, undergo shifts of approximately 15 nm to the red side of the spectrum. Here, diminishing S_11_ transitions throughout the titrations indicate strong electronic coupling between the *p*CNDs and HiPCO SWCNTs.

**Fig. 6:**
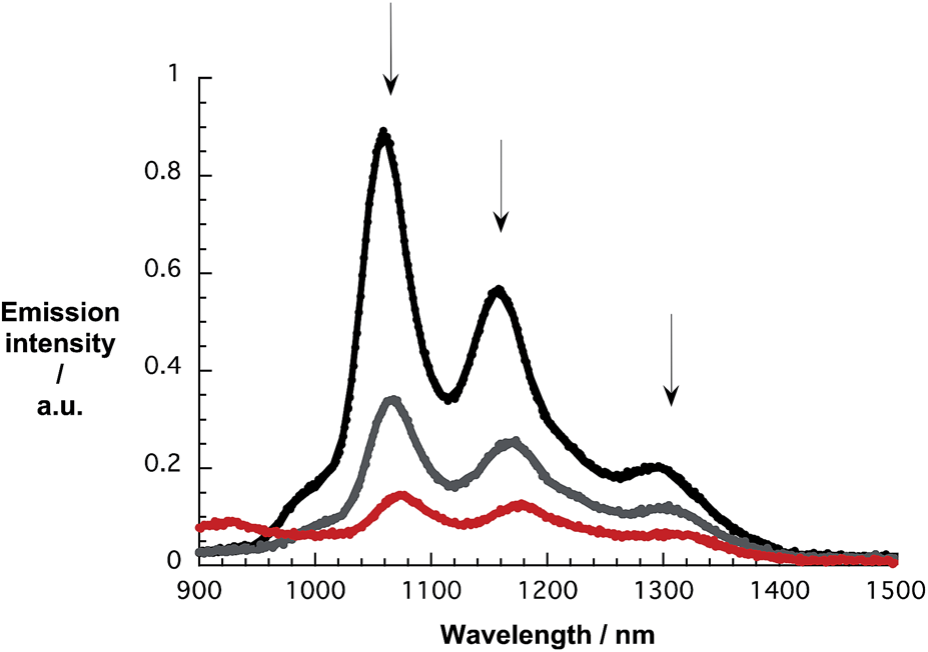
NIR emission spectra of CoMoCAT SWCNT/PVBTA upon excitation at 650 nm, recorded during sequential addition of *p*CND in D_2_O at room temperature – arrows refer to the course of addition.

As a complement to the absorption titrations, we used the same suspensions and conducted near-infrared emission measurements to monitor changes in the SWCNT-centered emission. As an example, the emission spectra of pure CoMoCAT SWCNT/PVBTA in D_2_O and with increasing concentrations of *p*CNDs excited at 650 nm are shown in Fig. 6. The maxima at 1058, 1158, and 1295 nm resemble the electronic S_11_ transitions of (7,5), (7,6), and (9,5) SWCNTs. Notably, the addition of *p*CNDs leads to quantitative but also unselective quenching of the emission. A 15 to 20 nm red shift evolves during the quenching, a trend that is in sound agreement with what is seen in the absorption titrations. In particular, the aforementioned peaks now appear at 1073, 1177, and 1315 nm. The corresponding 3D-photoluminescence plots are shown in Fig. S14.† Similar observations were obtained in complementary experiments with HiPCO SWCNTs – Fig. S15 and S16.†

To verify these observations, we performed reversed titration experiments, in which we started with a given *p*CND concentration and added CoMoCAT SWCNT/PVBTA sequentially. In this case, a gradual increase of the SWCNT-related transitions was observed, accompanied by a noticeable blue shift during the titration – Fig. S13.†

**Fig. 7:**
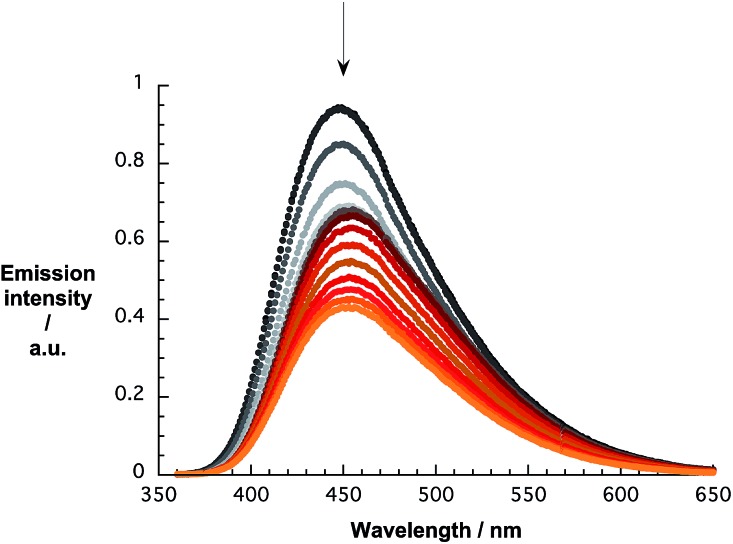
Emission spectra of *p*CND during the course of a titration with CoMoCAT SWCNT/PVBTA in D_2_O at room temperature – arrow refers to the course of addition.

The quenching of the *p*CND emission in the range around 450 nm during the reverse titrations gives rise to several interesting effects. Overall, the emission shifts from 446 to 458 and back to 453 nm, as shown in Fig. 7. A more profound analysis of the emission evolution was performed by fitting the spectra with an asymmetric double sigmoidal function.

**Fig. 8:**
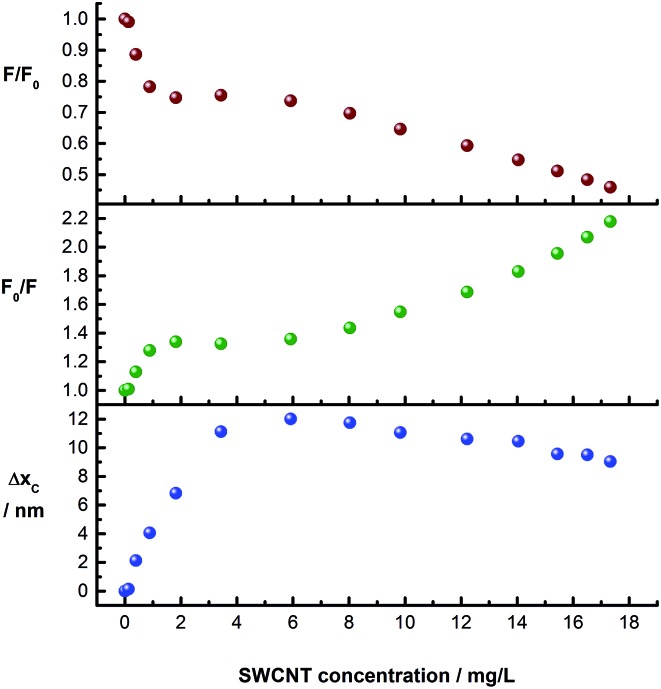
Evolution of the emission spectra of *p*CND dependent on the amount of CoMoCAT SWCNT/PVBTA added in D_2_O at room temperature. Upper panel (*F*/*F*_0_): quenching of the emission intensity. Center panel (*F*_0_/*F*): corresponding Stern–Volmer plot. Lower panel (Δ*x*_C_): shift of the peak maximum.

The evolution of the *p*CND-related emission during the course of the titration with CoMoCAT SWCNT/PVBTA is shown in Fig. 8. The upper and the central plots show the quenching (*F*/*F*_0_ and *F*_0_/*F*) plotted against SWCNT/PVBTA concentration. Strong quenching is observed up to a SWCNT/PVBTA equivalent of 2 mg L^–1^, followed by a plateau up to a concentration of 6 mg L^–1^. Upon further addition, more fluorescence quenching at a different rate sets in. Interestingly, this quenching correlates well with the shift of the maximum. The lower plot in Fig. 8 shows the evolution of the peak location *versus* SWCNT/PVBTA concentration. Again, a strong red-shift of 12 nm (from 446 to 458 nm) was observed in the concentration range between 0 and 4 mg L^–1^. This shift is replaced by a weaker blue shift from a concentration of 8 mg L^–1^ onwards.

**Fig. 9:**
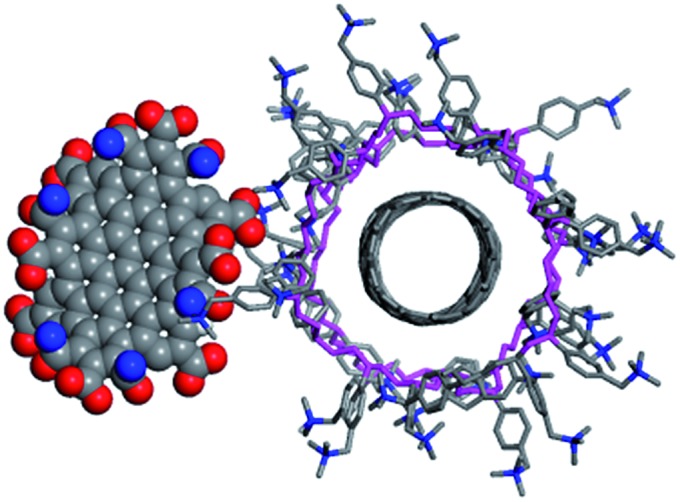
Structure of an SWCNT/PVBTA/*p*CND hybrid extracted from a molecular dynamics simulation.

These experiments clearly prove the interactions between both components. To obtain a qualitative structural model of the hybrid system, we performed molecular dynamics (MD) simulations (see computational details). Fig. 9 shows one structure of a SWCNT/PVBTA/*p*CND model system, taken from a molecular dynamics (MD) simulation. The negatively charged *p*CND interacts with the cationic polymer to form the hybrid material in solution. These simulations indicate that the observed electronic communication is not due to π–π-interactions.

**Fig. 10:**
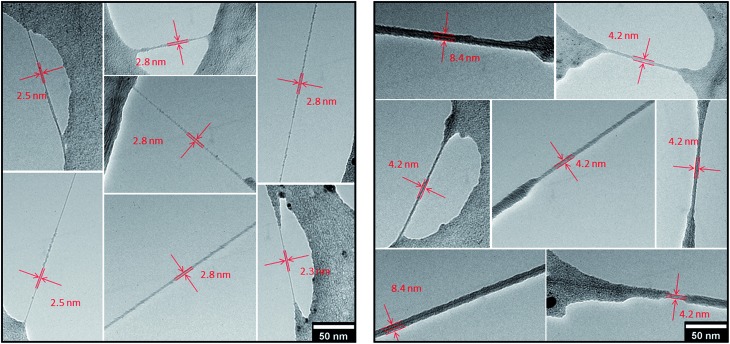
Representative TEM images of freestanding CoMoCAT SWCNT/PVBTA (left) and CoMoCAT SWCNT/PVBTA/*p*CND (right) on a Lacey carbon support film.

Transmission electron microscopic analyses of SWCNT/PVBTA after immobilization of *p*CND reveal individualized SWCNT/PVBTA/*p*CND throughout the entire TEM grid. Images documenting the individualization are shown in Fig. S17.† According to the models, the diameters of SWCNT wrapped with PVBTA are expected to be in a range between 2 and 3.5 nm, while those of the SWCNT/PVBTA/*p*CND assemblies should feature diameters between 4 and 5 nm. These diameters are in sound agreement with our TEM investigations. Both SWCNT/PVBTA and SWCNT/PVBTA/*p*CND were applied on Lacey carbon film supported TEM grids to obtain freestanding SWCNTs. As mentioned above, drying of the samples leads to the formation of thin polymer films, into which the SWCNT assemblies are incorporated. The TEM images in Fig. 10 show a series of micrographs of individual SWCNT/PVBTA (left part) and SWCNT/PVBTA/*p*CND (right part). From Fig. 10, diameters of 2.3–2.8 nm for SWCNT/PVBTA and ∼4.2 nm or ∼8.4 nm for individualized SWCNT/PVBTA/*p*CND and smaller bundles thereof, respectively, were determined.

Next, we probed the effect of addition of SWCNT/PVBTA on the emission lifetimes of *p*CND. Overall, the decays obtained by time-correlated single-photon counting (TCSPC), which spanned through a few tens of ns, were best fitted by triexponential functions to obtain *χ* ^2^ values with standard errors of less than 1 ± 0.01 to yield lifetimes of 2, 6, and 12 ns. Upon addition of SWCNT/PVBTA, all three lifetimes are reduced significantly, as shown in Table 1. Strikingly, the shortest lifetime begins to dominate when SWCNT/PVBTA is present. Lifetime measurements conducted with CoMoCAT and HiPCO SWCNT/PVBTA reveal similar trends – Fig. S18 and S19.†

**Table 1:** Emission lifetimes and their corresponding amplitudes obtained by TCSPC for *p*CND with different amounts of CoMoCAT SWCNT/PVBTA. Samples were excited at 403 nm and time profiles were fitted at 445 nm

	*τ*_1_ [ns]		*τ*_2_ [ns]		*τ*_3_ [ns]	
*p*CND	2.0	(26%)	6.0	(38%)	12	(36%)
*p*CND/SWCNT/PVBTA (1)	0.4	(49%)	2.4	(26%)	9.1	(24%)
*p*CND/SWCNT/PVBTA (2)	0.4	(48%)	2.4	(28%)	7.5	(24%)

**Fig. 11:**
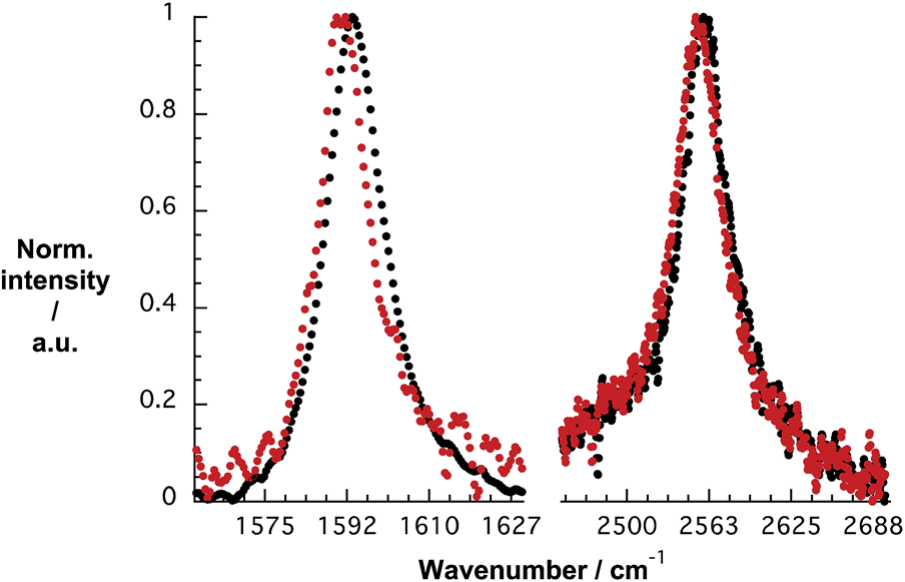
Normalized solid state Raman spectra (*λ*_ex_ = 1064 nm) of CoMoCAT SWCNT/PVBTA (black) and CoMoCAT SWCNT/PVBTA/*p*CND (red) with particular emphasis on the G-band and 2D-region.

To gain deeper insight into doping of SWCNTs on addition of *p*CND, we conducted complementary Raman experiments with SWCNT/PVBTA and SWCNT/PVBTA/*p*CND. The G^+^-band, as well as the 2D-band, show appreciable down-shifts upon adding *p*CND to both CoMoCAT and HiPCO SWCNT/PVBTA, suggesting that electrons are donated from the *p*CNDs to the SWCNTs – Fig. 11 and S20.† The observed G-band shift of 2.3 cm^–1^ from 1594.2 to 1591.9 cm^–1^ in the case of CoMoCAT SWCNT is very large for chemical doping of SWCNTs.^36^

**Fig. 12:**
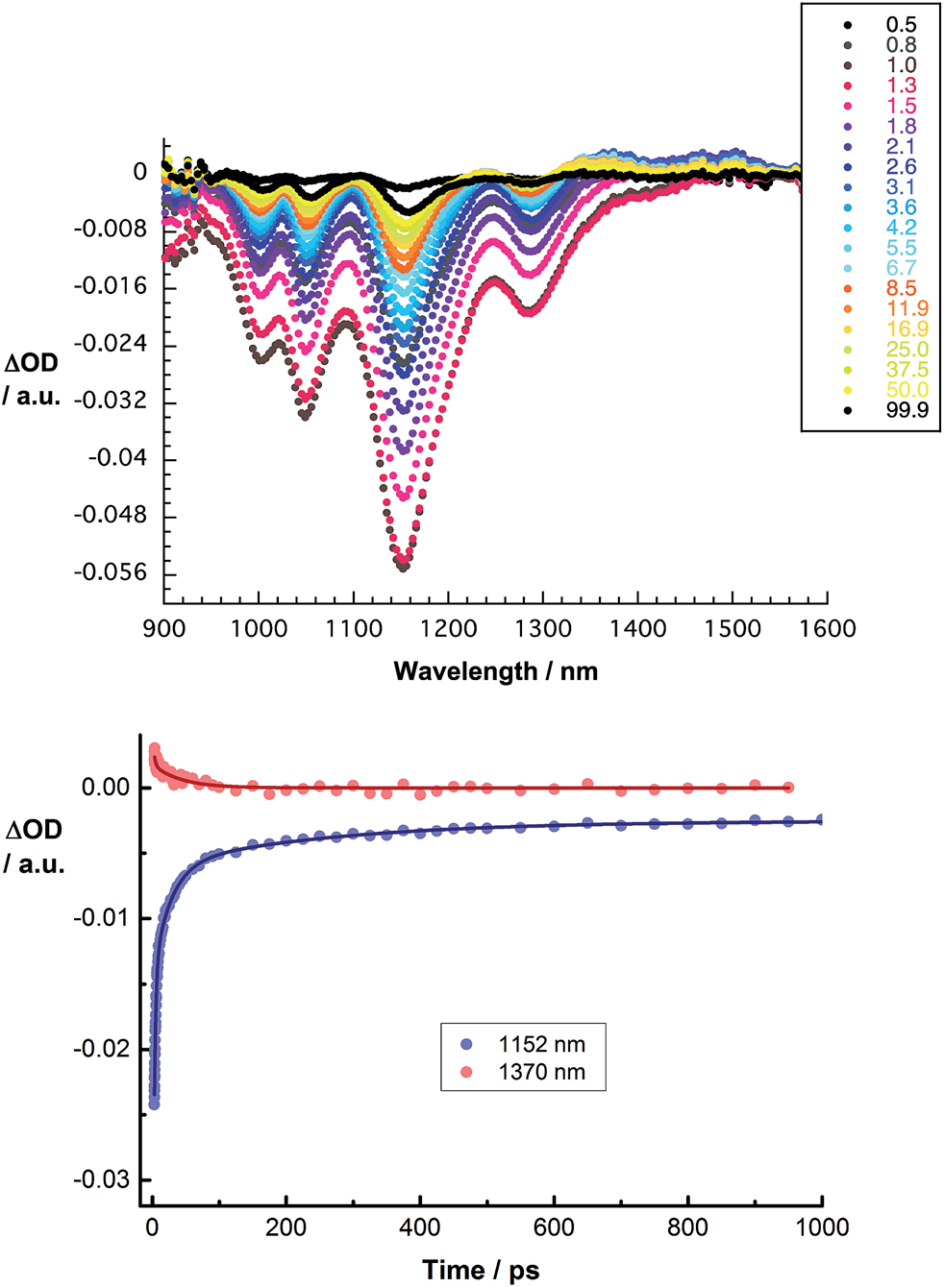
Upper part – differential absorption spectra obtained from femtosecond pump probe experiments (*λ*_ex_ = 387 nm) of CoMoCAT SWCNT/PVBTA with several time delays between 0.5 and 99.9 ps at room temperature. Lower part – corresponding time absorption profiles of the spectra shown above at 1152 nm (blue) and 1370 nm (red) monitoring the excited state decay.

We turned to femtosecond transient absorption spectroscopy to investigate the charge-transfer dynamics. Here, the excited state dynamics of SWCNT/PVBTA were investigated and compared with those of SWCNT/PVBTA/*p*CND. All samples were excited at 387 nm, where both *p*CNDs and SWCNTs feature appreciable ground state absorptions, or at 660 nm, where SWCNTs are selectively excited. Immediately upon photoexcitation of CoMoCAT SWCNT/PVBTA, a set of transient maxima at 1245 and 1364 nm and minima at 1000, 1048, 1152 and 1287 nm evolve, as shown in Fig. 12. These represent the excited-state features of CoMoCAT SWCNTs.^16^ The bleaching features in the NIR are reflections of the ground state bands, seen in the absorption experiments. The ground state recovery of CoMoCAT SWCNT/PVBTA takes place within a few hundred picoseconds and is characterized by three different lifetimes: 2, 25, and 260 ps.

**Fig. 13:**
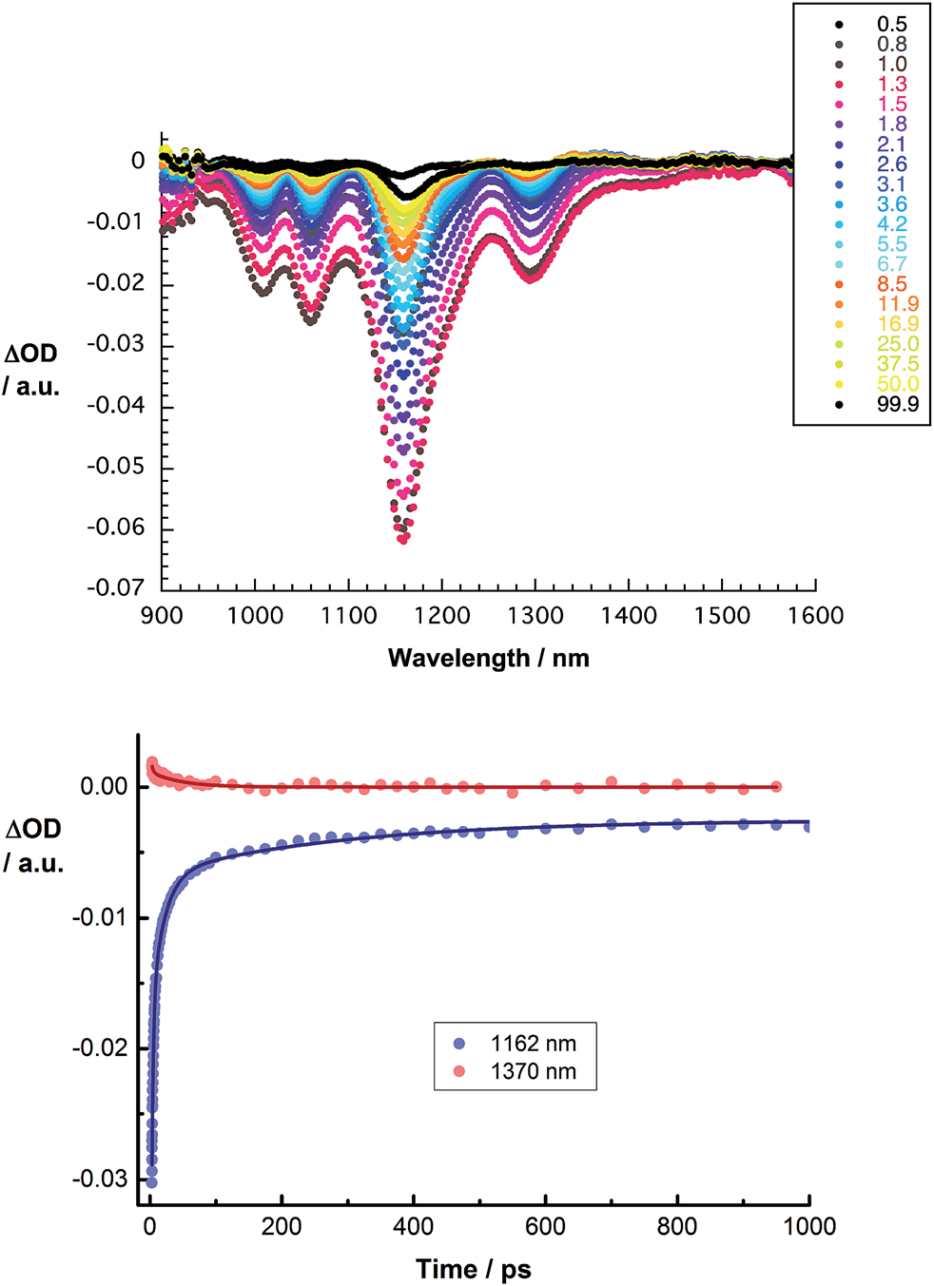
Upper part – differential absorption spectra obtained upon femtosecond pump probe experiments (*λ*_ex_ = 387 nm) of CoMoCAT SWCNT/PVBTA/*p*CND with several time delays between 0.5 and 99.9 ps at room temperature. Lower part – corresponding time absorption profiles of the spectra shown above at 1162 nm (blue) and 1370 nm (red) monitoring the excited state decay.

Several conclusions can be drawn from the transient absorption spectra of CoMoCAT SWCNT/PVBTA/*p*CND. In particular, an overall red shift of the aforementioned maxima to 1255 and 1370 nm and minima to 1010, 1059, 1161 and 1298 nm is observed in the initial spectrum, as shown in Fig. 13. Relative to CoMoCAT SWCNT/PVBTA, this reflects the formation of a complex between CoMoCAT SWCNT/PVBTA and *p*CND, in which a substantial delocalization of charge density shifts the SWCNT-centered bleaching to lower energies – *vide supra*. As time progresses, the dominant minima, which correspond to the ground state bleaching in the NIR, shift from, for example, 1010 and 1296 nm to 1005 and 1293 nm. In addition, the 1255 nm maximum appears with an additional lifetime component. Considering that all of these features are assigned to reduced CoMoCAT SWCNTs – as established in previous work based on complementary spectroelectrochemical and photophysical assays^25^ – we infer the formation of a charge separated state. In the context of oxidized *p*CND, we turned to its electrochemical oxidation, as shown in Fig. S21 and S22 in the ESI.† To this end, upon oxidation, the ground state absorption at around 350 nm decreases in intensity, while new absorption features between 400 and 600 nm are noted. In the transient absorption spectra, this spectral region is, however, dominated by SWCNT-centered absorptions.^37^ The correspondingly formed reduced CoMoCAT SWCNTs and oxidized *p*CNDs recombine with a lifetime of 105 ± 11 ps.^20^ The lifetimes for HiPCO SWCNT/PVBTA/*p*CND – Fig. S24† – are 20 ± 5 ps for the charge separated state lifetime.

## Conclusions

In summary, we have successfully decorated SWCNTs with *p*CNDs. The *p*CNDs used in this work are rather monodisperse with very large bandgaps on the order of 3.2 eV (Fig. S3†) and served as electron donors. Immobilization of the negatively charged *p*CNDs onto SWCNTs was enabled by means of wrapping with a positively charged polymer, namely PVBTA.

The herein presented results all suggest effective charge transfer between SWCNTs and *p*CNDs in the ground and excited states. The first evidence came from steady state absorption and emission titrations. Upon addition of *p*CND to the SWCNT/PVBTA hybrid, appreciable red shifts and decreases of the SWCNT-related absorption features were observed. These coincided with a noticeable quenching and shifting of the *p*CND- and SWCNT-related emissions. Shifted *p*CND and SWCNT absorptions/emissions point to a redistribution of charge density, that is, from the *p*CNDs to the SWCNTs. Further corroboration for this hypothesis came from rather strong downshifts of the G-band in Raman experiments with SWCNT/PVBTA and SWCNT/PVBTA/*p*CND. Full charge separation, that is, formation of a charge separated state with reduced CoMoCAT SWCNTs and oxidized *p*CNDs, was found in pump probe experiments.

## Experimental section

The preparation of pressure carbon nanodots (*p*CND) has been described previously.^29^ CoMoCAT SWCNTs SG 76, enriched with (7,6) SWCNTs, were obtained from Sigma Aldrich. Super-purified HiPCO SWCNTs were obtained from Unidym.

SWCNT/PVBTA hybrids were prepared by mixing 0.1 mg SWCNT and 1 mg PVBTA in 5 mL D_2_O. The mixture was sonicated using bath-type sonication (37 kHz, power: 100%) at room temperature for 30 min and then centrifuged at 15 kG for 10 min. The supernatant liquid was used for spectroscopic analysis. For the spectrophotometric titrations, SWCNT suspensions with optical densities of ∼0.2 at 850 and 950 nm for CoMoCAT and HiPCO, respectively, were prepared. The SWCNT suspensions were divided into two portions. To one portion, solid *p*CND was added to obtain a concentration of 20 mg L^–1^. For the titrations, the *p*CND-containing portion was added stepwise to the pure SWCNT/PVBTA suspension.

Ultrasonication, used for the preparation of SWCNT suspensions, was carried out with a Elmasonic P120 52 (330 W) from ELMA. Steady-state absorption measurements were carried out with a Cary 5000 UV/Vis/NIR-spectrometer (Varian). Steady-state fluorescence emission measurements were performed with a FluoroMax®-3. Time-correlated single-photon counting and near-infrared steady state emission spectroscopy was performed with a FluoroLog®-3 spectro-fluorometer (Horiba). All spectra were corrected for the instrument response. Transmission electron microscopy was performed with a TEM 912 Omega (Zeiss). Femtosecond transient absorption studies were carried out using a Helios transient absorption pump/probe system from Ultrafast Systems with laser pulses fed by a CPA-2101 from Clark-MXR Inc. Raman spectra were recorded using a FT-Raman RFS 100 system from Bruker with a Ge detector using a 1064 nm wavelength Nd-YAG laser for excitation.

Computational model systems were built based on a 100 ps molecular dynamics run of a (7,5)-SWCNT/PVBTA system in aqueous solution, with 40 chloride counterions. This simulation was performed at ambient pressure and 300 K (NPT ensemble) using the COMPASS force field, as implemented in Forcite Plus.^38^ The simulation box was sized 90 × 94 × 85 Å^3^ with periodic boundary conditions in all three dimensions. The *p*CND model was based on our previous work.^38^ To obtain a negatively charged dot, eight amide groups were replaced by carboxylates.

The molecular electrostatic potential was calculated with EMPIRE, using the semiempirical AM1 Hamiltonian and 1D periodic boundary conditions along the tube axis.^39,40,41^ The figures were made with VMD.^42^
