# Supplementary material for: A carbon–carbon hybrid – immobilizing carbon nanodots onto carbon nanotubes
Source: Chem Sci. 2015 Aug 20;6(12):6878–85. doi: 10.1039/c5sc02728d (PMC5510013; doi:10.1039/c5sc02728d)
Supplement: Supplementary file 1 [file SC-006-C5SC02728D-s001.pdf]

Electronic Supplementary Material (ESI) for Chemical Science.
This journal is © The Royal Society of Chemistry 2015

Supporting Information to

A Carbon-Carbon Hybrid – Immobilizing Carbon Nanodots and Carbon Nanotubes

Volker Strauss, Johannes T. Margraf, Timothy Clark, Dirk M. Guldi

Structures S2
Steady State Absorption Spectroscopy S3
Photoluminescence S4
Raman Spectroscopy S6
Transmission Electron Microscopy S7
Steady State Absorption Titrations S8
SWCNT Photoluminescence Quenching with pCND S9
Transmission Electron Microscopy of SWCNT/PVBTA/pCND S10
Fluorescence Lifetime Measurements (TCSPC) S11
Steady State Emission Titrations S12
Spectroelectrochemical Data (Absorption and Emission) S13
Femtosecond Transient Absorption S14

Structure

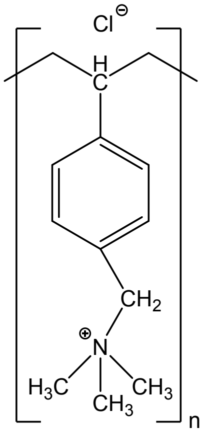

Figure S1. Structure of poly(4-vinylbenzyl trimethylamine) (PVBTA)

Steady State Absorption Spectroscopy

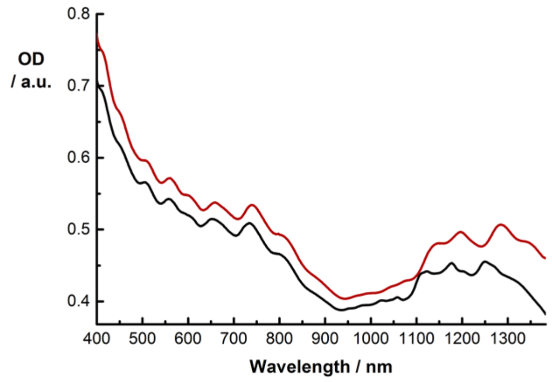

Figure S2. Absorption spectra of **HiPCO SWCNT** dispersed with SDBS (2wt%, black) and PVBTA (2wt%, red) in D_2O.

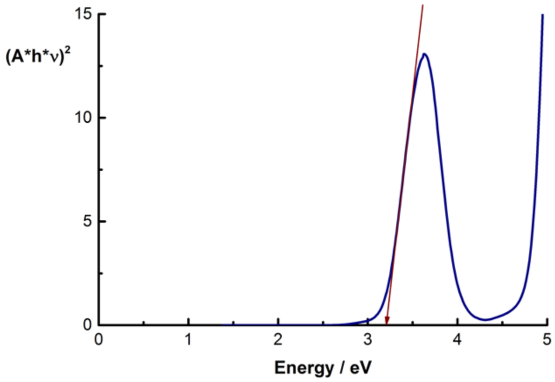

Figure S3. Tauc plot of **pCND** in H_2O.

Photoluminescence

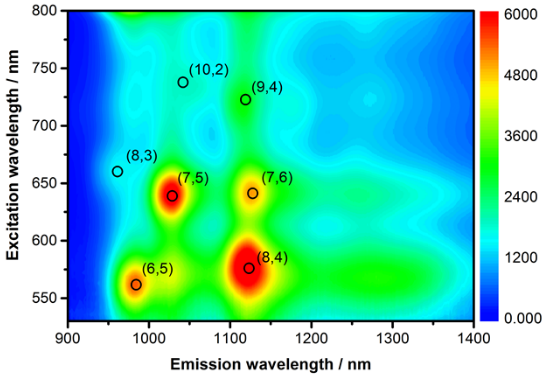

Figure S4. 3D-Photoluminescence plot of **CoMoCAT SWCNT/SDBS** in D_2O at room temperature with peak assignment.

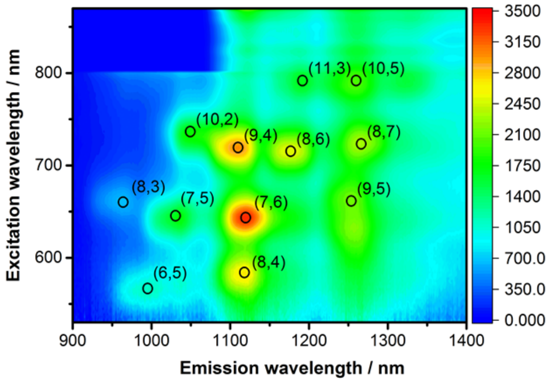

Figure S5. 3D-Photoluminescence plot of **HiPCO SWCNT/SDBS** in D_2O at room temperature.

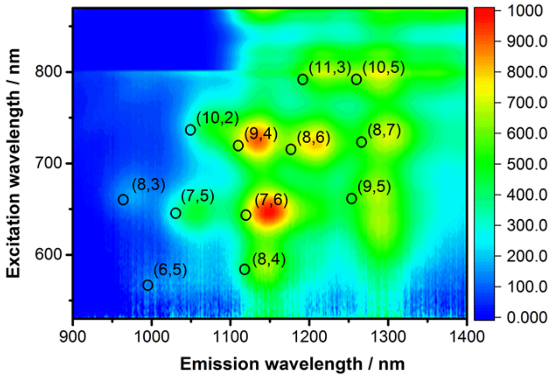

Figure S6. 3D-Photoluminescence plot of **HiPCO SWCNT/PVBTA** in D_2O at room temperature.

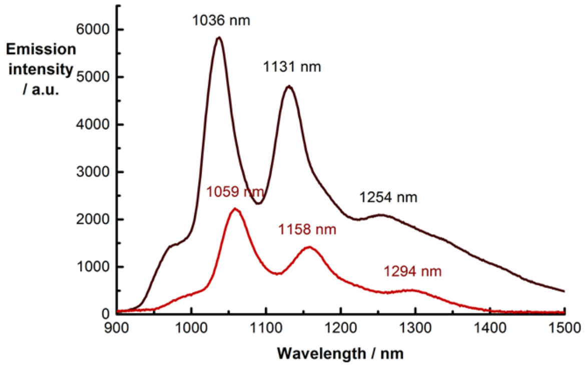

Figure S7. NIR emission spectra of dispersions of **CoMoCAT SWCNT/SDBS** (wine) and **CoMoCAT SWCNT/PVBTA** (red) with equal optical density at the 650 nm excitation wavelength in D_2O at room temperature.

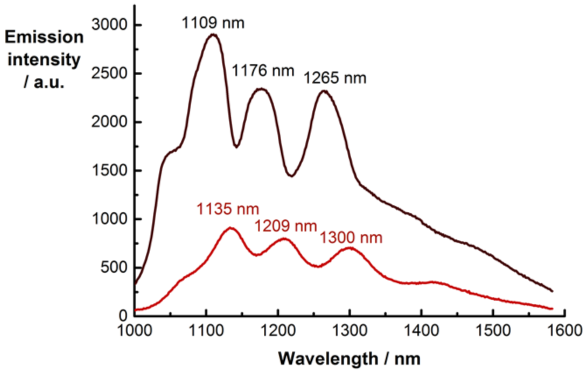

Figure S8. NIR emission spectra of dispersions of **HiPCO SWCNT/SDBS** (wine) and **HiPCO SWCNT/PVBTA** (red) with equal optical density at the 725 nm excitation wavelength in D_2O at room temperature.

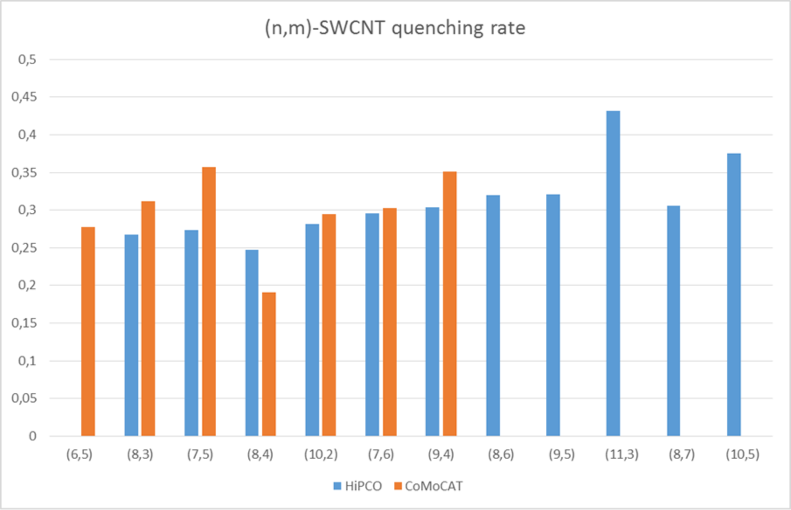

Figure S9. Quenching rates relative to the SDBS reference dispersions for the different SWCNT species present in **HiPCO SWCNT/PVBTA and** (blue) and **CoMoCAT SWCNT/PVBTA** (orange) dispersions.

Raman Spectroscopy

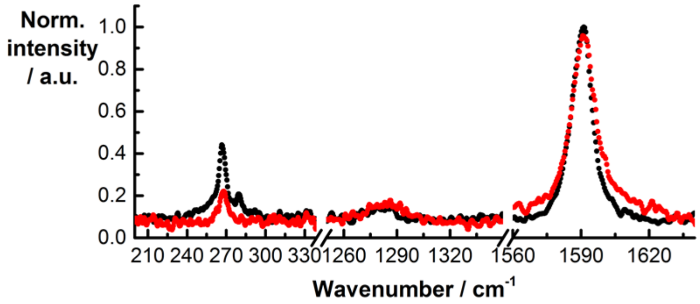

Figure S10. Normalized solid state Raman spectra (λ_{ex} = 1064nm) of **HiPCO SWCNT/SDBS** (black) and **HiPCO SWCNT/PVBTA** (red) with particular emphasis on the RBM- (left), D-band (center), and G-band (right) regions.

Transmission Electron Microscopy

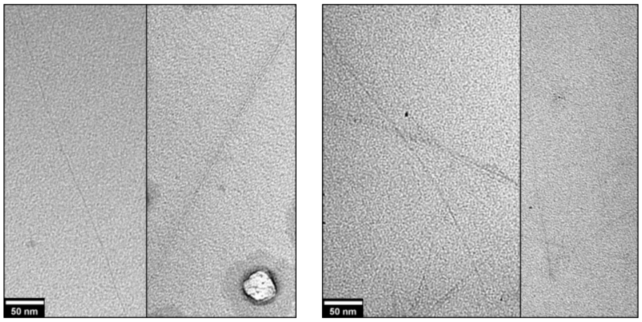

Figure S11. Representative TEM images of **CoMoCAT SWCNT/PVBTA** (left) and **HiPCO SWCNT/PVBTA** (right) on an ultrathin carbon support film.

Steady State Absorption Titrations

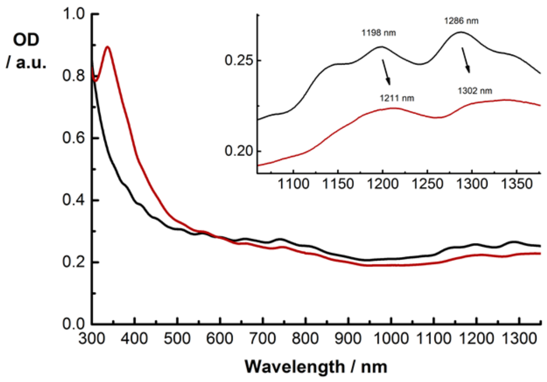

Figure S12. Absorption spectra of **HiPCO SWCNT/PVBTA** (black) and of **HiPCO SWCNT/PVBTA/pCND** (red) in D_2O at room temperature.

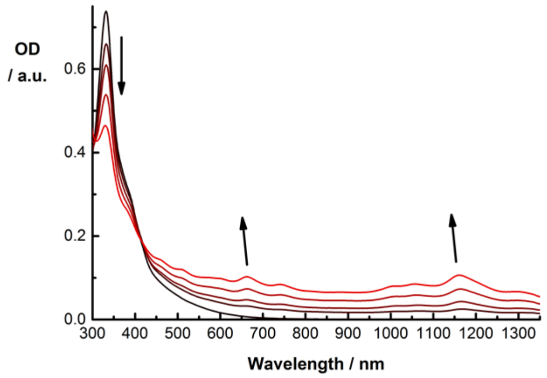

Figure S13. Absorption spectra recorded in the course of sequential addition of **pCND** to **CoMoCAT SWCNT/PVBTA** in D_2O at room temperature.

SWCNT Photoluminescence Quenching with pCND

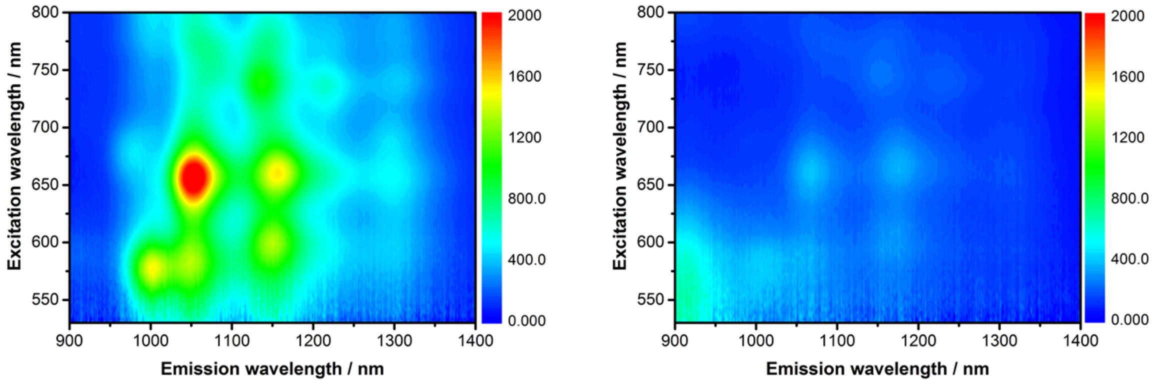

Figure S14. 3D-Photoluminescence plots of **CoMoCAT SWCNT/PVBTA** (left) and **CoMoCAT SWCNT/PVBTA/pCND** (right) in D_2O at room temperature.

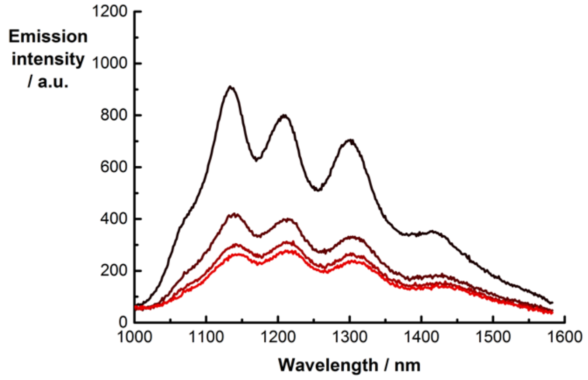

Figure S15. NIR emission spectra of **HiPCO SWCNT/PVBTA** upon excitation at 725 nm recorded during sequential addition of **pCND** in D_2O at room temperature.

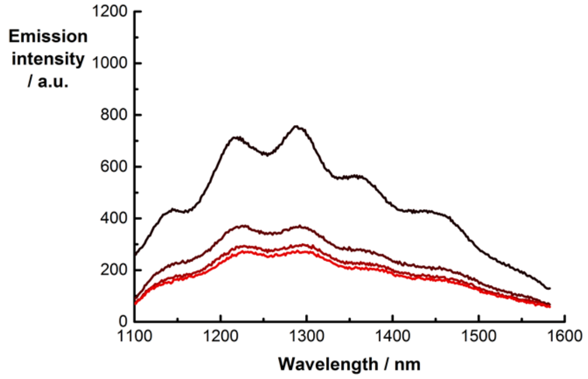

Figure S16. NIR emission spectra of **HiPCO SWCNT/PVBTA** upon excitation at 800 nm recorded during sequential addition of **pCND** in D_2O at room temperature.

Transmission Electron Microscopy of SWCNT/PVBTA/pCND

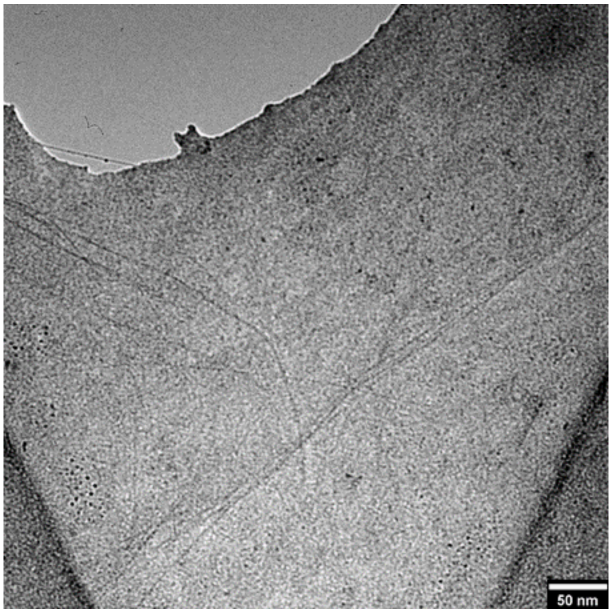

Figure S17. Representative TEM images of **CoMoCAT SWCNT/PVBTA/pCND** on an Lacey carbon support film.

Fluorescence Lifetime Measurements (TCSPC)

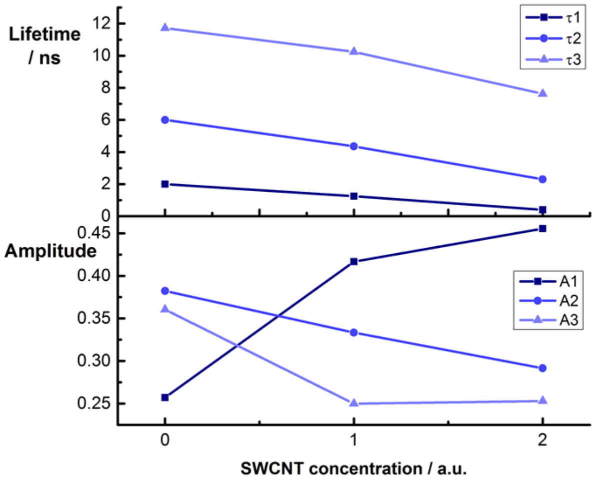

Figure S18. Evolution of the emission lifetimes and their corresponding amplitudes of **pCND** as a function of the amount of **CoMoCAT SWCNT/PVBTA** obtained by TCSPC. Samples were excited at 403 nm and time profiles were measured at 445 nm.

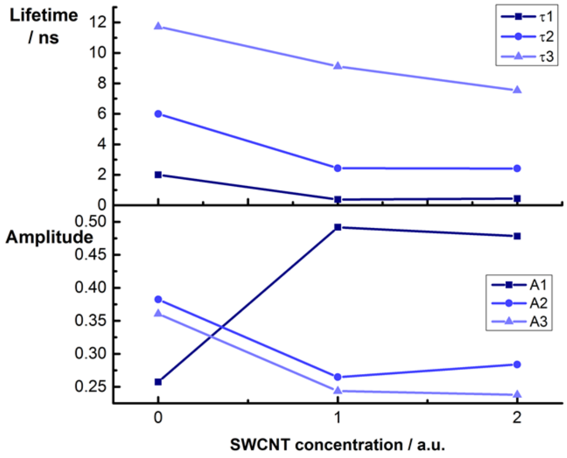

Figure S19: Evolution of the emission lifetimes and their corresponding amplitudes of **pCND** as a function of the amount of **HiPCO SWCNT/PVBTA** obtained by TCSPC. Samples were excited at 403 nm and time profiles were measured at 445 nm.

Steady State Emission Titrations

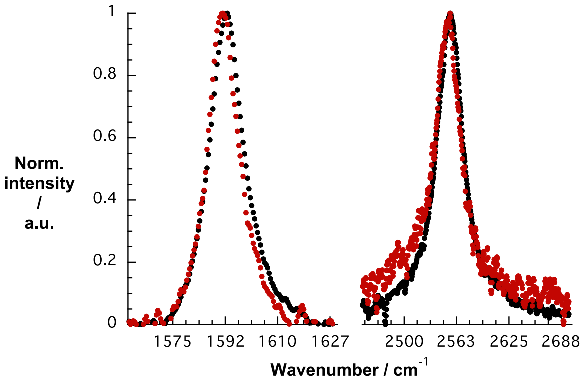

Figure S20. Normalized solid state Raman spectra (λ_{ex} = 1064nm) of **HiPCO SWCNT/PVBTA** (black) and **SWCNT/PVBTA/pCND** (red) with particular emphasis on the G-band and 2D-region.

Spectroelectrochemical Data (Absorption and Emission)

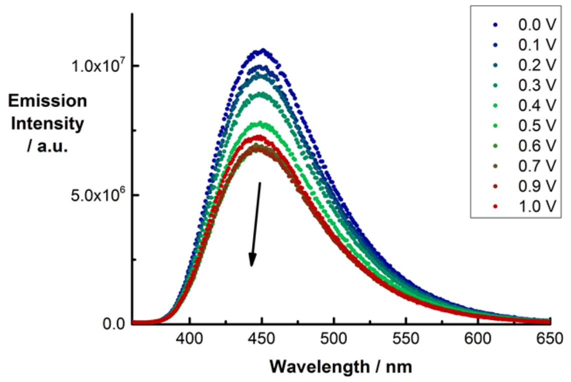

Figure S21: Emission spectra of **pCND** upon stepwise oxidation from 0 – 1 V in H_2O with 0.1 M $TBAPF_6$ as supporting electrolyte.

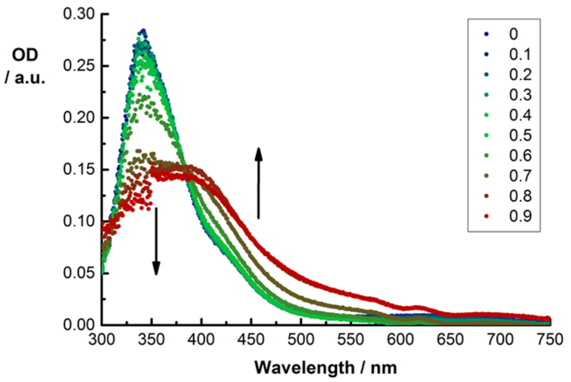

Figure S22: Absorption spectra of **pCND** upon stepwise oxidation from 0 – 1 V in H_2O with 0.1 M $TBAPF_6$ as supporting electrolyte.

Femtosecond Transient Absorption

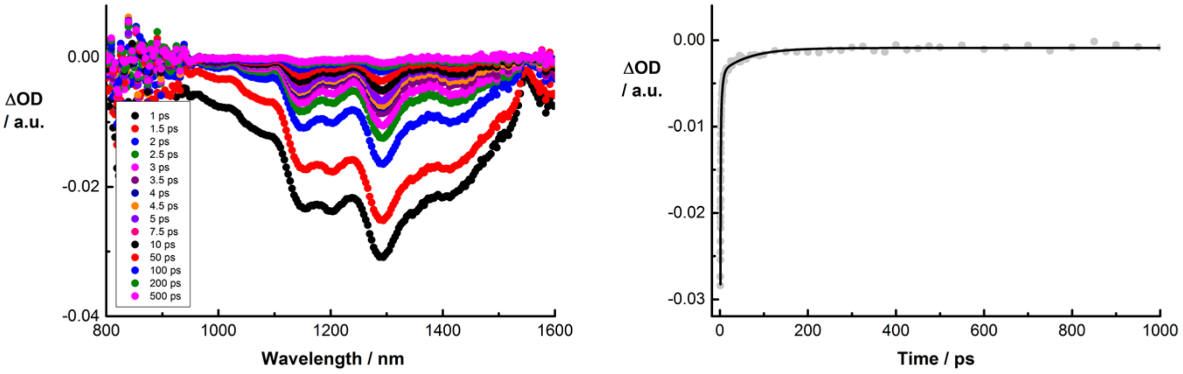

Figure S23. Left: Differential absorption spectra obtained upon femtosecond pump probe experiments (λ_{ex} = 387 nm) of **HiPCO SWCNT/PVBTA** with several time delays between 1.5 and 500 ps at room temperature. Right: Corresponding time absorption profile of the spectra shown on the left at 1300 nm monitoring the excited state decay.

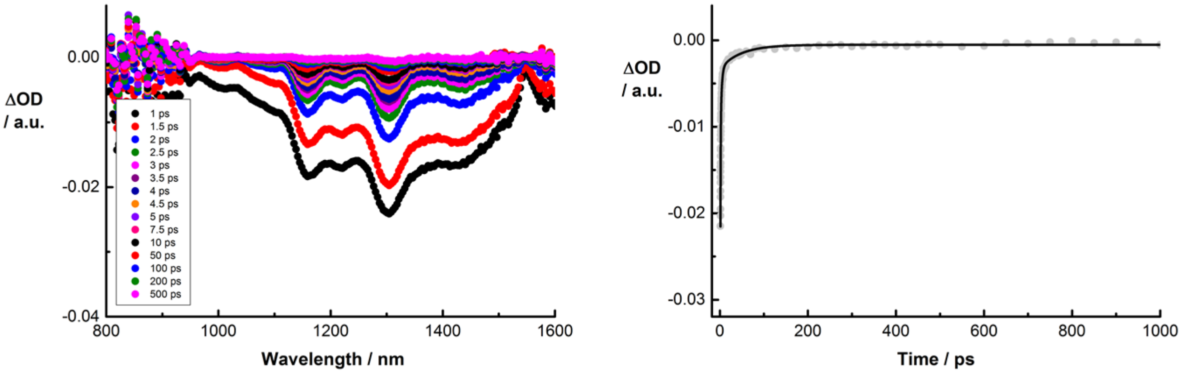

Figure S24. Left: Differential absorption spectra obtained upon femtosecond pump probe experiments (λ_{ex} = 387 nm) of **HiPCO SWCNT/PVBTA/pCND** with several time delays between 1.5 and 500 ps at room temperature. Right: Corresponding time absorption profile of the spectra shown on the left at 1300 nm monitoring the excited state decay.
